# Traditional Chinese medicines and natural products targeting immune cells in the treatment of metabolic-related fatty liver disease

**DOI:** 10.3389/fphar.2023.1195146

**Published:** 2023-06-09

**Authors:** Zhen Li, Hao Ouyang, Junfeng Zhu

**Affiliations:** Department of Liver, Yueyang Hospital of Integrated Traditional Chinese and Western Medicine, Shanghai University of Traditional Chinese Medicine, Shanghai, China

**Keywords:** metabolic-related fatty liver disease, immune cells, traditional Chinese medicine prescription, natural product, herb component, treatment

## Abstract

MAFLD stands for metabolic-related fatty liver disease, which is a prevalent liver disease affecting one-third of adults worldwide, and is strongly associated with obesity, hyperlipidemia, and type 2 diabetes. It encompasses a broad spectrum of conditions ranging from simple liver fat accumulation to advanced stages like chronic inflammation, tissue damage, fibrosis, cirrhosis, and even hepatocellular carcinoma. With limited approved drugs for MAFLD, identifying promising drug targets and developing effective treatment strategies is essential. The liver plays a critical role in regulating human immunity, and enriching innate and adaptive immune cells in the liver can significantly improve the pathological state of MAFLD. In the modern era of drug discovery, there is increasing evidence that traditional Chinese medicine prescriptions, natural products and herb components can effectively treat MAFLD. Our study aims to review the current evidence supporting the potential benefits of such treatments, specifically targeting immune cells that are responsible for the pathogenesis of MAFLD. By providing new insights into the development of traditional drugs for the treatment of MAFLD, our findings may pave the way for more effective and targeted therapeutic approaches.

## 1 Introduction

MAFLD is characterized by the accumulation of fat in the liver, which can lead to inflammation and scarring of the liver over time (Marques et al., 2023). It is closely linked to obesity, type 2 diabetes, high blood pressure, and dyslipidemia (abnormal levels of fats in the blood) (Gutierrez-Cuevas et al., 2021). The redefinition of non-alcoholic fatty liver disease (NAFLD) to MAFLD reflects a shift in the understanding of this condition as being part of a larger metabolic disease spectrum rather than a standalone liver condition (Eslam et al., 2020). This new definition also aims to simplify the diagnosis and management of the disease (Eslam et al., 2020). The proposed criteria for the diagnosis of MAFLD include evidence of fatty liver on imaging or histology, along with one of the following three criteria: overweight or obesity, presence of type 2 diabetes, or evidence of metabolic dysregulation (such as dyslipidemia or high blood pressure) (Eslam et al., 2020). While further research is needed to fully understand the implications of this new definition, it is hoped that it will improve diagnosis and treatment of this increasingly common chronic liver disease. MASH stands for nonalcoholic steatohepatitis, which refers to the inflammation and damage to liver cells resulting from the progression of MAFLD (Wu et al., 2022a). This includes the presence of a heterogeneous fatty liver, liver cell necrosis, and inflammatory reactions (Wu et al., 2022a).

Morbidity and mortality associated with MAFLD can vary depending on factors such as the severity of the disease, underlying metabolic disorders, and the presence of comorbidities (Aghemo et al., 2022). However, studies have shown that MAFLD increases the risk of developing liver-related complications such as MASH, cirrhosis, and hepatocellular carcinoma (HCC) (Marengo et al., 2016). It also increases the risk of developing cardiovascular diseases, which are the leading cause of death in patients with MAFLD (Gutierrez-Cuevas et al., 2021). According to recent studies, the global prevalence of MAFLD is estimated to range from 20% to 30% (Younossi et al., 2016). The liver-specific mortality rate among patients with MAFLD is reported to be higher than that of the general population (Younossi et al., 2016). The total mortality rate among patients with MAFLD is also reported to be higher than that of the general population, with cardiovascular disease being a major contributor to mortality (Chen et al., 2022a). Better understanding of the underlying molecular mechanisms of MAFLD will be critical for developing new therapeutic approaches to treat and prevent the complications of this disease.

The activation of innate immune cells (e.g., macrophages, neutrophils, monocytes, dendritic cells (DCs), T lymphocytes, B lymphocytes, natural killer (NK) cells, natural killer T (NKT) cells, as well as mast cells) can result in phagocytosis of cell debris and foreign antigens, release of pro-inflammatory cytokines, and production of reactive oxygen species (ROS) and reactive nitrogen species (RNS) (Kountouras et al., 2023). These events can lead to hepatocellular injury, cellular stress, and damage to the extracellular matrix (ECM) (Kountouras et al., 2023). The activation of adaptive immune cells can further exacerbate the inflammatory response and contribute to the development of liver fibrosis (Hu et al., 2020). In addition to the immune cells, other immune-related molecules and signaling pathways are also involved in the pathogenesis of MAFLD/MASH. Toll-like receptors (TLRs) can recognize pathogen-associated molecular patterns (PAMPs) and damage-associated molecular patterns (DAMPs) and activate the innate immune response (An et al., 2020; Park et al., 2021; Khanmohammadi and Kuchay, 2022). Nuclear factor κB (NF-κB) is a key transcription factor that regulates the expression of pro-inflammatory genes and is activated by TLR signaling, as well as other factors such as ROS and RNS (Kawai and Akira, 2007; Blaser et al., 2016). The inflammasome, a multiprotein complex that activates caspase-1 and promotes the secretion of pro-inflammatory cytokines such as interleukin (IL)-1β and IL-18, has also been implicated in the development of MASH (Du et al., 2019). In summary, the liver plays a critical role in the immune response, and the dysregulation of this response can contribute to the development and progression of MAFLD/MASH (Zhang et al., 2021a). Understanding the complex interactions between immune cells, signaling molecules, and the liver microenvironment will be crucial for developing effective therapies for these disorders.

Traditional Chinese medicine (TCM) prescription refers to a combination of several herbs or natural ingredients that are formulated to treat a specific health condition (Zhu, 2022). The prescription is often a unique blend of herbs and other natural ingredients in specific proportions which have been used for centuries in TCM (He et al., 2021). Natural products refer to remedies or supplements that are made from naturally occurring substances such as plants, minerals, or animal parts (Harvey et al., 2015). These products can be derived from various sources including herbs, fruits, vegetables, and other botanicals (Zhang et al., 2020a). Herb components are the active ingredients present in herbs (Al-Ishaq et al., 2019). These components may include alkaloids, flavonoids, terpenes, and other compounds that contribute to the therapeutic effects of the herb (Dasari et al., 2022). Herb components are often extracted from herbs and used in TCM prescriptions or natural products (Dasari et al., 2022). In summary, TCM prescription is a specific combination of herbs and natural ingredients used to treat a particular health condition, while natural products may contain one or more natural substances including herbs. Herb components are the individual active ingredients present in herbs that contribute to their therapeutic effects.

In this comprehensive review, we have examined the various functions of immune cells in MAFLD and explored the potential therapeutic benefits of TCM prescriptions, natural products and herb components for treating MAFLD by targeting immune cells. Furthermore, we have discussed the underlying mechanisms of these treatments, particularly with respect to immune cells.

## 2 Factors contributing to MAFLD

**FIGURE 1:**
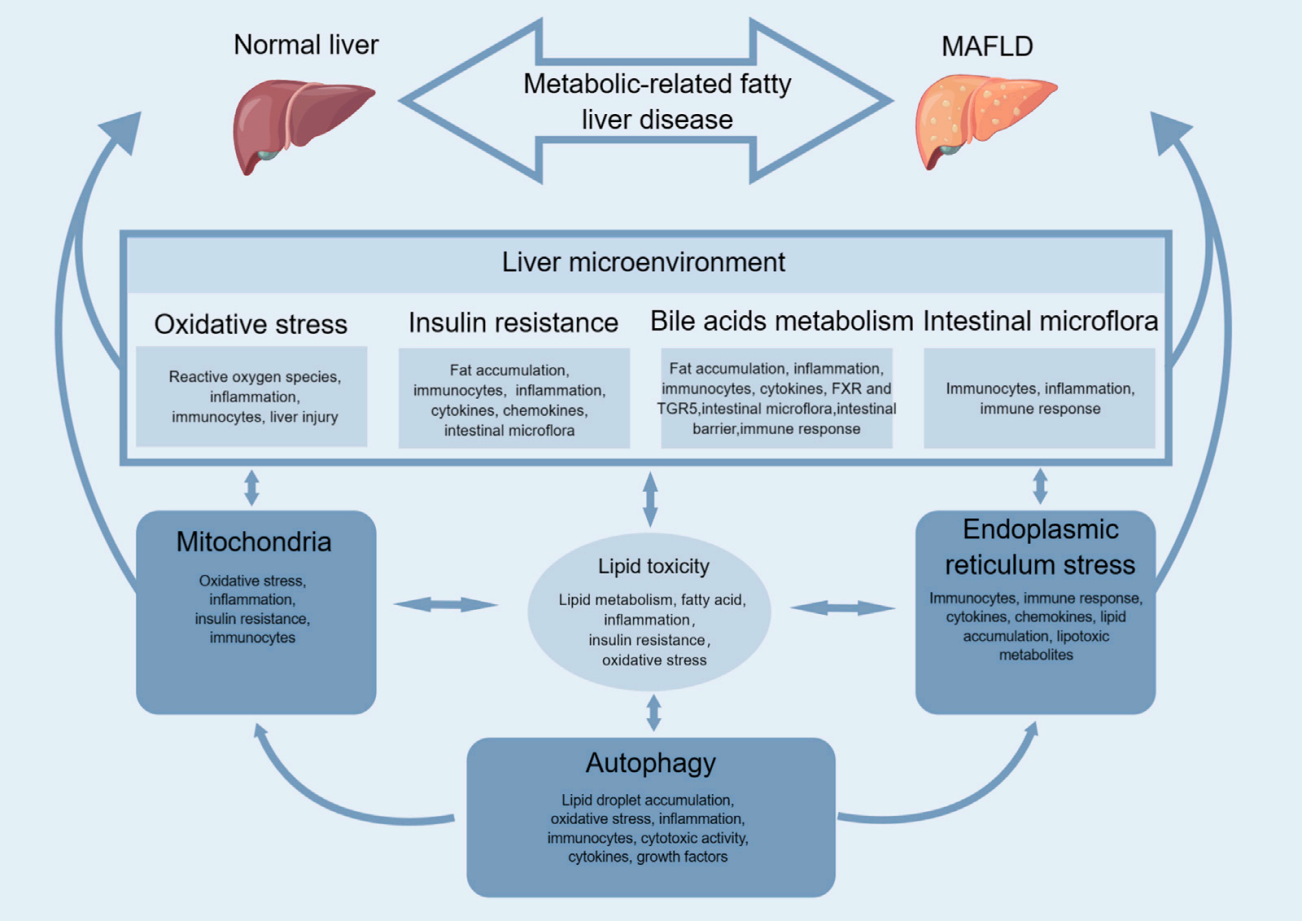
Mechanism of MAFLD.

There are multiple contributing factors, such as lipid toxicity, mitochondria, ER stress, autophagy, oxidative stress (OS), insulin resistance, bile acids metabolism, intestinal microflflora, etc., that result in the accumulation of fatty acids in hepatocytes. This accumulation causes damage to liver cells and ultimately leads to the formation of MAFLD (as illustrated in Figure 1).

### 2.1 Lipid toxicity

Lipid toxicity and MAFLD have a close relationship since lipid toxicity is the primary factor contributing to the development of MAFLD. Lipid toxicity refers to the accumulation of excess lipids within cells or tissues, leading to cellular damage and dysfunction (Weinberg, 2006). In the case of MAFLD, excessive lipid accumulation in the liver leads to hepatocyte damage, inflammation, and eventually, liver disease (Abulikemu et al., 2023). The liver plays a crucial role in regulating lipid metabolism, including the synthesis, oxidation, and storage of fat (Alves-Bezerra and Cohen, 2017). When there is an excess of lipids in the liver, it can lead to impaired liver function, insulin resistance, and OS, all of which contribute to the development and progression of MAFLD (Badmus et al., 2022). Several factors can lead to the accumulation of lipid in the liver, such as dietary habits, genetics, and lifestyle factors such as lack of physical activity and alcohol consumption (Manne et al., 2018). Therefore, it is possible to reduce the progression of MAFLD and improve overall liver health by reducing lipid toxicity in the liver.

### 2.2 Mitochondria

Mitochondria, the cellular organelles responsible for energy production, play a crucial role in the pathogenesis of MAFLD. Studies have shown that dysfunctional mitochondria contribute to the development of MAFLD by promoting OS, inflammation, and insulin resistance. Immune cells, particularly macrophages, also play a critical role in the progression of MAFLD (Ramanathan et al., 2022). In response to mitochondrial dysfunction and hepatocellular injury, immune cells infiltrate the liver and release pro-inflammatory cytokines, exacerbating inflammation and fibrosis (Fu et al., 2022). The relationship between mitochondrial dysfunction and immune cells in MAFLD is complex and bidirectional. Dysfunctional mitochondria can activate immune cells, leading to an increase in pro-inflammatory cytokines and OS (Ahmed et al., 2019). In turn, immune cell infiltration and activation can further impair mitochondrial function, perpetuating the cycle of inflammation and injury (Field et al., 2020). Modulating the immune response and improving mitochondrial function are potential therapeutic targets for MAFLD.

### 2.3 Endoplasmic reticulum stress

The ER is a cellular organelle involved in various functions such as protein synthesis, lipid metabolism, and calcium storage. In recent years, research has shown that ER stress and dysfunction are linked to the development of metabolic disorders including MAFLD and MASH (Yuan et al., 2020). Immune cells play a crucial role in the pathogenesis of MAFLD and MASH (de Oliveira et al., 2019). Various immune cells such as macrophages and T cells infiltrate the liver and contribute to liver inflammation and fibrosis (Peiseler et al., 2022). The ER is involved in regulating the immune response through several mechanisms, including the processing and presentation of antigens, cytokine production, and maintenance of calcium homeostasis (Oakes and Papa, 2015). Recent studies have suggested that ER stress can activate the unfolded protein response (UPR), which can either promote or suppress immune cell activation and function (Grootjans et al., 2016). For example, UPR activation can induce the expression of pro-inflammatory cytokines and chemokines that attract immune cells to the liver (Zhang et al., 2021b). However, prolonged UPR activation can also induce the expression of anti-inflammatory cytokines and regulatory T cells, which can limit liver inflammation (Salminen et al., 2020). In addition, ER stress can affect the function of immune cells themselves (Bettigole and Glimcher, 2015). For instance, ER stress-induced autophagy can enhance the antigen presentation capacity of DCs, leading to increased T cell activation (Poncet et al., 2021). However, excessive ER stress can impair T cell proliferation and survival (Cotte et al., 2018). Finally, the ER is involved in lipid metabolism, and ER stress and dysfunction can lead to the accumulation of lipids and the generation of lipotoxic metabolites that can activate immune cells and promote liver inflammation (Mastrototaro and Roden, 2021). Conclusively, the relationship between ER stress, immune cells, and MAFLD is complex and multifaceted. Further research is needed to fully understand the mechanisms underlying this relationship and to develop novel therapeutic strategies for MAFLD.

### 2.4 Autophagy

The relationship between autophagy and MAFLD immune cells is complex and not yet fully understood. Autophagy is a cellular process that involves the degradation of damaged or dysfunctional cellular components, including pathogens and cellular debris, by lysosomal enzymes (Mizushima and Komatsu, 2011). It has been suggested that impaired autophagy may contribute to the development and progression of MAFLD, as it can lead to the accumulation of lipid droplets, OS, and inflammation in hepatocytes (Chen and Lin, 2022). Immune cells also play a crucial role in the pathogenesis of MAFLD, as they are involved in the processes of inflammation, fibrosis, and hepatic steatosis (Torre et al., 2021). Studies have shown that there is a dynamic interplay between autophagy and immune cells in the development and progression of MAFLD (Wang et al., 2019a). For instance, autophagy can modulate the activation and differentiation of immune cells, such as macrophages and T cells, by regulating the release of pro-inflammatory cytokines and chemokines (Germic et al., 2019). Moreover, recent evidence suggests that autophagy can directly regulate the function of immune cells in the liver, such as NK cells and NKT cells, by modulating their cytotoxic activity and cytokine production (Loucif et al., 2022). On the other hand, immune cells can also regulate the autophagic activity in hepatocytes, as they secrete cytokines and growth factors that can either promote or inhibit autophagy (Gukovskaya et al., 2017). Further studies are needed to fully understand this relationship and to develop new therapeutic interventions for MAFLD that target both autophagy and immune cells.

### 2.5 Oxidative stress

OS is a state in which there is an imbalance between the production of ROS and the body’s ability to detoxify them (Delli Bovi et al., 2021). It is a common feature in MAFLD due to the accumulation of fat in the liver, leading to increased inflammation and ROS production (Guo et al., 2021). The liver plays a crucial role in the immune system as it is the site of immune cell recruitment and activation (Corazza et al., 2009). MAFLD may lead to alterations in immune cell function and impair the liver’s ability to respond to infections and other insults (Corazza et al., 2009; Barchetta et al., 2020). Immune cells, such as macrophages and T cells, are involved in the development of MAFLD and can also contribute to OS through the release of inflammatory cytokines and ROS (De Jesus et al., 2020). There is evidence to suggest that OS can modulate immune cell activity, whereby excessive ROS production can cause immune dysfunction and impair the immune response (Sun et al., 2020). Conversely, activation of certain immune cells, such as NK cells, can help to control ROS production and prevent liver damage (Papp et al., 2016). In the end, OS, MAFLD, and immune cells are closely interlinked, with each factor impacting the others. Strategies aimed at reducing OS, such as using antioxidants, and targeting immune cell infiltration hold promise for preventing or treating MAFLD.

### 2.6 Insulin resistance

Insulin resistance and MAFLD have a complex relationship with immunocytes. Insulin resistance is known to be associated with chronic low-grade inflammation, which can lead to the accumulation of fat in the liver and contribute to the development of MAFLD (Sakurai et al., 2021). Immunocytes, such as macrophages, neutrophils, and T cells, are involved in the inflammatory response and play a role in the pathogenesis of MAFLD (Miyake et al., 2010). In MAFLD, there is an increased infiltration of immunocytes, particularly macrophages, into the liver (Kolakowski et al., 2022). These macrophages release pro-inflammatory cytokines and chemokines that further promote inflammation and insulin resistance (Olefsky and Glass, 2010). In addition, T cells have also been shown to contribute to liver inflammation in MAFLD by producing pro-inflammatory cytokines (Peiseler et al., 2022). Recent studies have suggested that there is also a bidirectional relationship between MAFLD and immunocytes (Peiseler et al., 2022). For example, it has been shown that immunocytes themselves can contribute to insulin resistance and worsen MAFLD (El-Arabey and Abdalla, 2022). Additionally, the gut microbiome, which can influence immune function, has also been implicated in the development of both insulin resistance and MAFLD (Liu et al., 2016). Ultimately, the relationship between insulin resistance, MAFLD, and immunocytes is complex and involves multiple pathways and mechanisms (Liu et al., 2016). Further research is needed to better understand this relationship and develop effective treatments for these conditions.

### 2.7 Bile acids metabolism

Bile acids are essential for the digestion and absorption of dietary fats in the small intestine. They are also signaling molecules that regulate several physiological processes, including glucose and lipid metabolism, energy expenditure, and inflammation. In MAFLD, the accumulation of fat in the liver triggers an inflammatory response, characterized by the infiltration of immune cells, such as macrophages and T cells (Zhang et al., 2021a). These cells release pro-inflammatory cytokines, which exacerbate liver damage and promote fibrosis (Brenner et al., 2013). Bile acids play a dual role in this process (Chen et al., 2022b). On the one hand, they can act as anti-inflammatory agents by binding to specific receptors, such as FXR and TGR5, on immune cells and hepatocytes (Chiang and Ferrell, 2020). Activation of these receptors inhibits the production of pro-inflammatory cytokines and promotes the expression of anti-inflammatory genes (Haggerty et al., 2023). On the other hand, bile acids can also promote inflammation by disrupting the gut microbiota and increasing the permeability of the intestinal barrier (Haggerty et al., 2023). This leads to the translocation of bacterial products, such as lipopolysaccharides (LPS), into the bloodstream, which triggers an immune response and promotes liver inflammation (Carpino et al., 2020). To conclude, the balance between pro- and anti-inflammatory effects of bile acids is critical for the development and progression of MAFLD. Understanding this delicate interplay between bile acids and immune cells may lead to novel therapeutic approaches for this prevalent disease.

### 2.8 Intestinal microflora

There is a growing body of research suggesting that intestinal microflora may play a role in the development and progression of MAFLD. The intestinal microflora refers to the collection of microorganisms that live within our gut, including bacteria, viruses, fungi, and other microorganisms (Pluta et al., 2021). The interaction between these microorganisms and the immune cells in our gut is thought to play a key role in the development and progression of MAFLD (Park et al., 2021). Studies have suggested that changes in the composition of the intestinal microflora can lead to an increase in the levels of pro-inflammatory cytokines in the gut, which can lead to the activation of immune cells and the development of MAFLD (Park et al., 2021). For example, studies have shown that certain types of immune cells, such as T-cells, can be activated by the presence of certain types of bacteria in the gut, leading to inflammation and damage to the liver (Im et al., 2012). The intestinal microbiota plays an important role in modulating the immune response by facilitating the development of regulatory T cells, which can help to suppress inflammation and prevent autoimmune diseases (Behary et al., 2021). All in all, while more research is needed to fully understand the relationship between intestinal microflora, immune cells, and MAFLD, there is mounting evidence to suggest that the composition of the intestinal microflora plays a key role in the development and progression of this condition.

## 3 Immune system in MAFLD

The innate immune system is the first line of defense against foreign substances (Gandhi and Vliagoftis, 2015). It includes physical barriers such as skin and mucous membranes, as well as cells such as phagocytes and NK cells that can recognize and destroy pathogens (Parkin and Cohen, 2001). The adaptive immune system is activated when the innate immune system is unable to eliminate an invading pathogen (Geremia et al., 2014). This system targets specific antigens and creates a memory of the pathogen for future encounters. Humoral immunity is mediated by B cells, which produce antibodies that recognize and neutralize specific antigens (Cancro and Tomayko, 2021). Cellular immunity is mediated by T cells, which can directly kill infected cells or coordinate the immune response by releasing cytokines that activate other immune cells (Russo and Brogan, 2014). Immunoreactive substances such as antibodies, complement proteins, and leukocytes play a crucial role in the immune response (Mi et al., 2018). Antibodies bind to specific antigens and can neutralize or mark pathogens for destruction by phagocytes (Fischman and Ofran, 2018). Complement proteins can also mark pathogens for destruction and can activate other components of the immune system (Stephan et al., 2012). Leukocytes are involved in all aspects of the immune response, from recognizing and eliminating pathogens to coordinating the immune response (Abdallah et al., 2021). Overall, the immune system is a complex network of organs, cells, and molecules that work together to protect the body from infection and maintain a healthy internal environment.

In the early stages of MAFLD, hepatic steatosis and inflammation are primarily driven by innate immune cells such as KCs/macrophages, neutrophils, and DCs (Lamadrid et al., 2021). KCs/macrophages play a critical role in the initiation and progression of MAFLD by releasing pro-inflammatory cytokines and chemokines, promoting OS, and inducing hepatocyte apoptosis (Remmerie et al., 2020). Neutrophils are also involved in the early stages of MAFLD by producing ROS, which contribute to OS and inflammation (Aguilar et al., 2023). DCs are antigen-presenting cells that present antigens to T cells and play a crucial role in regulating T cell-mediated immune responses (Lv et al., 2020). In MAFLD, DCs promote the differentiation of pro-inflammatory Th1 and Th17 cells, contributing to the development of liver inflammation and fibrosis (Ye et al., 2020). In the later stages of MAFLD, adaptive immune cells such as T cells and B cells become more prevalent and contribute to the progression of liver injury (Hu et al., 2020). CD4^+^ T cells, particularly Th1 and Th17 cells, play a critical role in the pathogenesis of MAFLD by producing pro-inflammatory cytokines such as interferon-gamma (IFN-γ) and IL-17, respectively (Zhou et al., 2022). CD8^+^ T cells also contribute to liver injury in MAFLD by inducing hepatocyte apoptosis and producing cytotoxic molecules such as perforin and granzyme B (Wang et al., 2019b). B cells, which produce antibodies against specific antigens, are also involved in MAFLD (Barrow et al., 2021). Recent studies have shown that B cells can contribute to the development of liver fibrosis by producing pro-inflammatory cytokines and activating hepatic stellate cells (HCSs) (Gaul et al., 2021). Besides, NK cells, NKT cells and mast cells are also involved in MAFLD, with NKT cells playing a pathogenic role by promoting inflammation and fibrosis (Kennedy et al., 2021; Yang et al., 2021; Olveira et al., 2023).

In conclusion, immune cells play a crucial role in the development and progression of MAFLD. The dysregulation of innate and adaptive immune responses contributes to the pathogenesis of MAFLD and its complications. Understanding the mechanisms of immune cell involvement in MAFLD may lead to the development of new therapeutic strategies for this disease.

### 3.1 Macrophages

The relationship between macrophages and MAFLD is complex and multifaceted. Macrophages are immune cells that play a critical role in the development and progression of MAFLD, which is characterized by the accumulation of fat in the liver, inflammation, and damage to liver cells (Remmerie et al., 2020). Macrophages infiltrate the liver in response to various stimuli, including excess fat, alcohol consumption, and viral infection (Skuratovskaia et al., 2020). They are involved in both the initiation and progression of MAFLD, promoting inflammation, fibrosis, and insulin resistance (Thibaut et al., 2022). In the early stages of MAFLD, macrophages secrete pro-inflammatory cytokines such as TNF-α and IL-6, which contribute to liver injury and promote the development of insulin resistance (Zhang et al., 2019). As the disease progresses, macrophages also contribute to the development of fibrosis by secreting profibrogenic factors such as transforming growth factor-beta (TGF-β)1 (Deng et al., 2022). Recent research has also highlighted the role of macrophage polarization in the pathogenesis of MAFLD (Kolakowski et al., 2022). In particular, a shift towards a pro-inflammatory M1 phenotype has been shown to be associated with the development of MAFLD and its progression to MASH, while a shift towards an anti-inflammatory M2 phenotype may have a protective effect (Xu et al., 2020).

**FIGURE 2:**
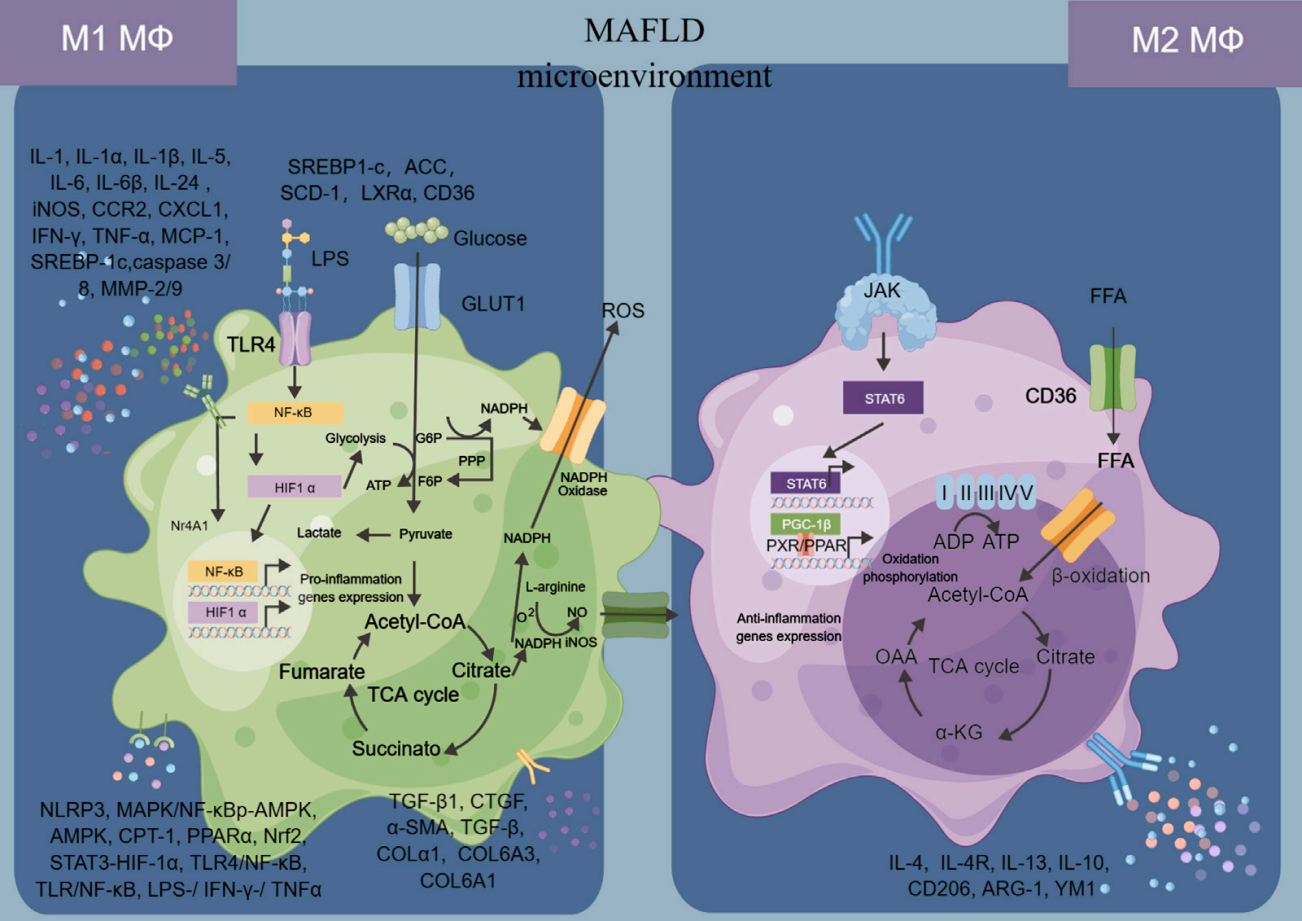
Macrophages in MAFLD.

In short, the relationship between macrophages and MAFLD is complex and dynamic, with macrophages playing a key role in both the development and progression of the disease (Figure 2). Understanding the interaction between macrophages and MAFLD is key to developing effective treatments for this condition. Some potential strategies include targeting inflammatory pathways or modulating the activity of macrophages in the liver.

### 3.2 Neutrophils

**FIGURE 3:**
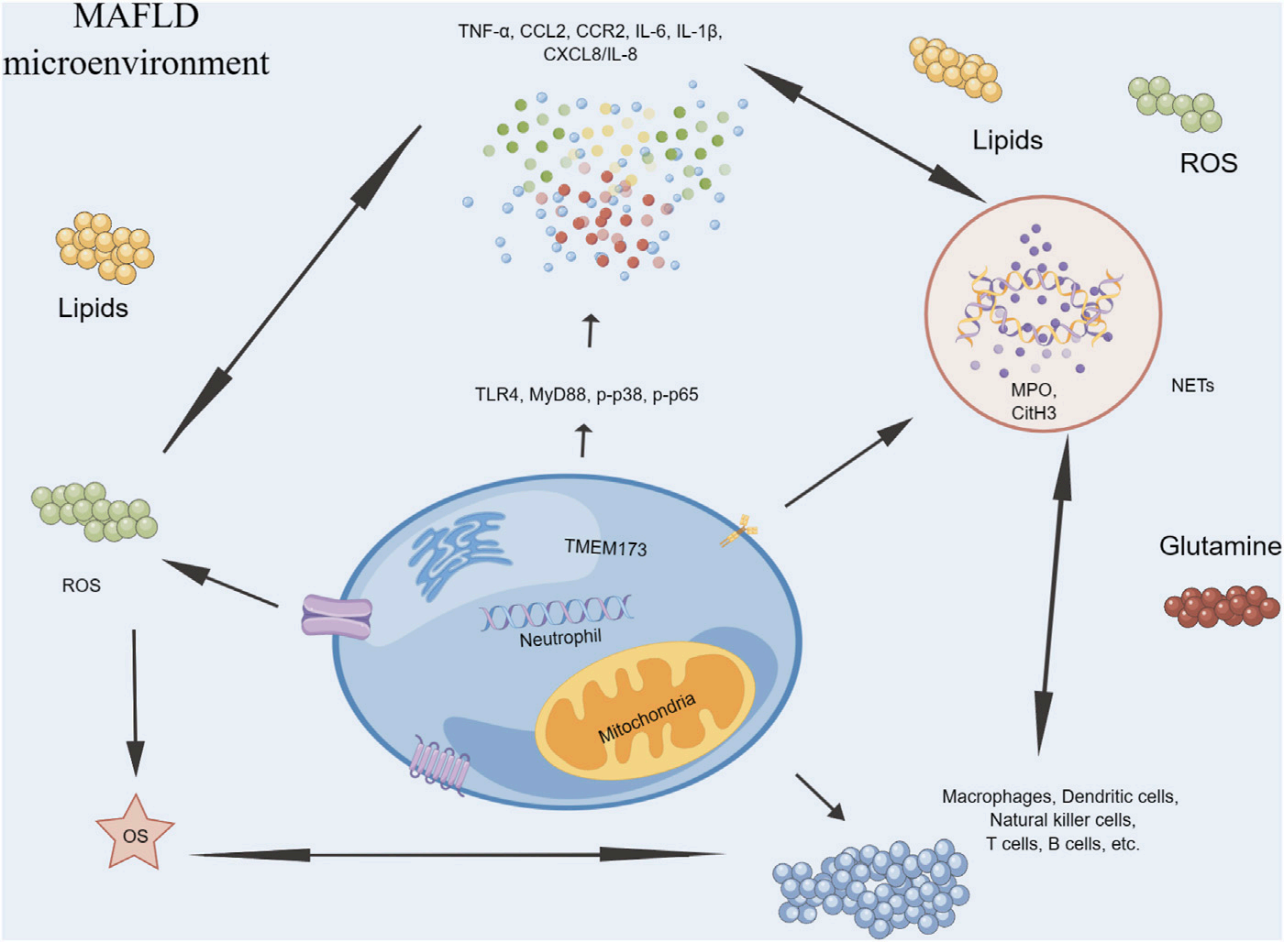
Neutrophils in MAFLD.

There is emerging evidence to suggest a relationship between neutrophils and MAFLD (Figure 3). During the early stages of MAFLD, neutrophils are one of the first immune cells to migrate towards the liver and become activated (Bourgonje et al., 2022). Once activated, neutrophils release ROS as part of their antimicrobial defense mechanisms (Ferreyra Solari et al., 2012). However, excessive production of ROS can lead to OS and tissue damage, contributing to the development of inflammation and liver fibrosis (Farzanegi et al., 2019). Furthermore, neutrophils also release cytokines and chemokines that attract other immune cells such as monocytes and macrophages, leading to a sustained inflammatory response in the liver (Mridha et al., 2017). This chronic inflammation can further exacerbate OS and tissue damage, ultimately leading to the progression of MAFLD to more severe stages such as MASH and cirrhosis (Luci et al., 2020). Additionally, recent research has suggested that neutrophil extracellular traps (NETs), a structure composed of DNA, histones, and antimicrobial peptides released by neutrophils, may also play a role in MAFLD (Wang et al., 2021a). NETs can activate innate immune cells such as macrophages, DCs, and NK cells, leading to the release of pro-inflammatory cytokines and chemokines, including IL-1β, TNF-α, IL-6, and CXCL8/IL-8 (Papayannopoulos, 2018). These mediators can further recruit and activate immune cells, exacerbating inflammation and tissue damage, contributing to the development and progression of MAFLD (Wang et al., 2021a).

Therefore, targeting neutrophils may be a potential therapeutic strategy in the early stages of MAFLD. For instance, drugs that inhibit neutrophil recruitment, activation, or NETosis could be developed to reduce inflammation and liver damage associated with MAFLD.

### 3.3 T cells

**FIGURE 4:**
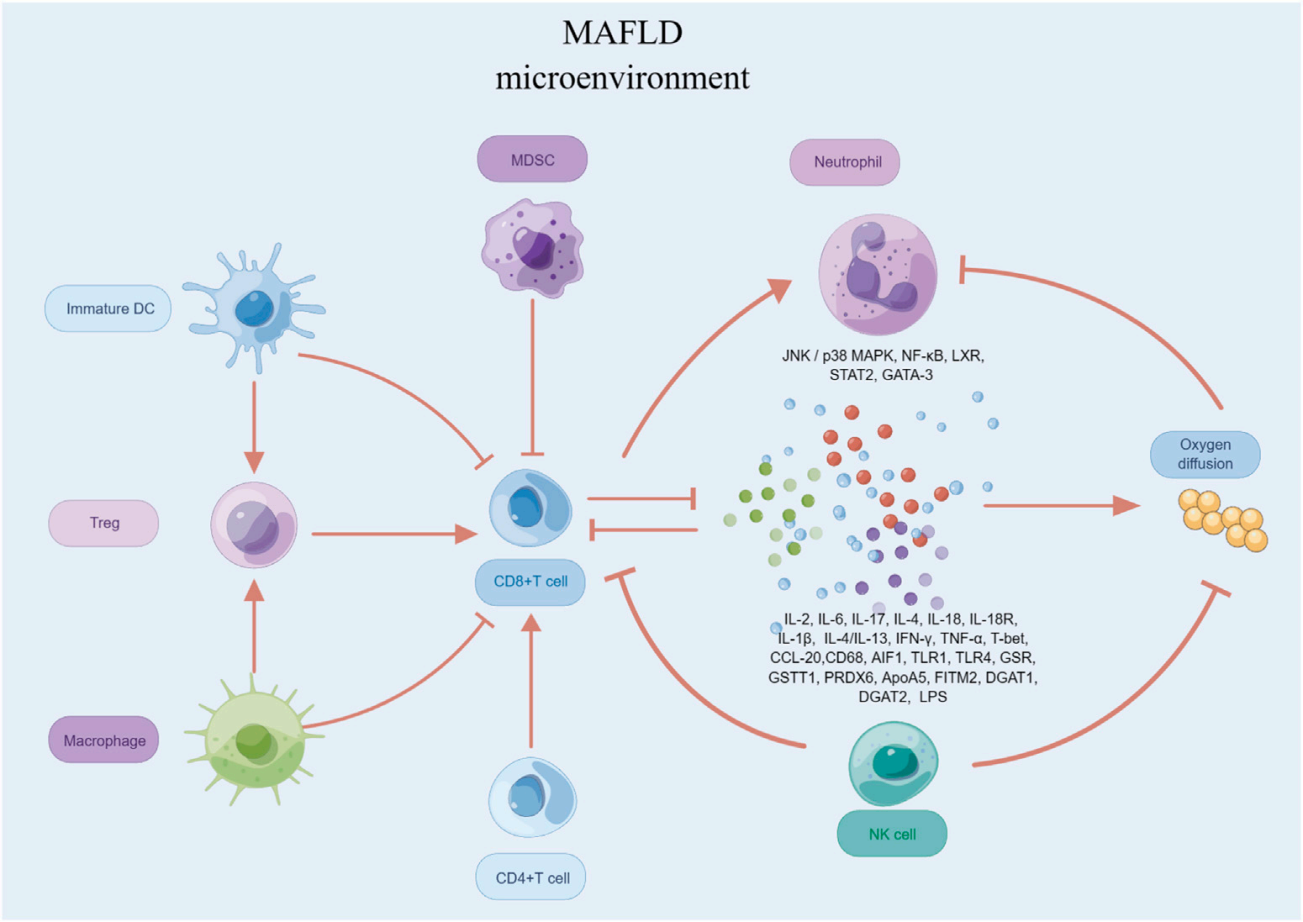
T cells in MAFLD.

T cells play a critical role in the development and progression of MAFLD (Figure 4). Studies have shown that T cells can contribute to the pathogenesis of MAFLD through various mechanisms, including inflammation, OS, and fibrosis (Wang et al., 2022a). In particular, studies have identified CD4^+^ T cells, also known as T helper cells, as playing a significant role in MAFLD (Ma et al., 2016). These cells can secrete pro-inflammatory cytokines, such as IL-2, IL-17, IFN-γ and TNF-α, which can contribute to liver damage and fibrosis. CD8^+^ T cells are involved in the killing of infected hepatocytes, but their activity can also lead to further liver damage and inflammation, wich secrete several pro-inflammatory cytokines, including IL-2, IL-6, IL-17, IFN-γ, and TNF-α (Plochg et al., 2022). Additionally, Tregs have been shown to play a protective role in MAFLD by suppressing inflammation and promoting liver regeneration (Wang et al., 2020).

In summary, the relationship between T cells and MAFLD is complex and multifactorial, and more research is needed to fully understand the underlying mechanisms and potential therapeutic targets for this disease.

### 3.4 B cells

As the disease progresses, chronic inflammation and OS in the liver cause damage to hepatocytes, which leads to the release of damage-associated molecular patterns (DAMPs) and microbial products into the liver microenvironment (Huang et al., 2015). These molecules activate immune cells such as B cells and T cells and promote the release of pro-inflammatory cytokines (Segura-Cerda et al., 2020). B cells are a type of adaptive immune cell that produce antibodies to recognize and neutralize invading pathogens (Wang et al., 2022b). In MAFLD, B cells are activated by DAMPs and produce autoantibodies against self-antigens in the liver (Faas and de Vos, 2020). These autoantibodies can further damage liver cells and activate other immune cells to exacerbate inflammation. In addition, B cells can also differentiate into plasma cells, which secrete large amounts of pro-inflammatory cytokines (such as IL-6 and TNF-α) and exacerbate the immune response (Rosser and Mauri, 2015). Recent studies have shown that B cells also play a role in regulating lipid metabolism in the liver (Postic et al., 2004). B cells have the ability to uptake and metabolize lipids, and their activation can lead to alterations in lipid metabolism that exacerbate steatosis and liver injury (Zhang et al., 2022).

In brief, the increased presence and activation of B cells in later stages of MAFLD contribute to the progression of liver injury by promoting inflammation, damaging liver cells, and altering lipid metabolism. Targeting B cells may be a promising therapeutic strategy to treat advanced MAFLD.

### 3.5 Natural killer (NK) cells

**FIGURE 5:**
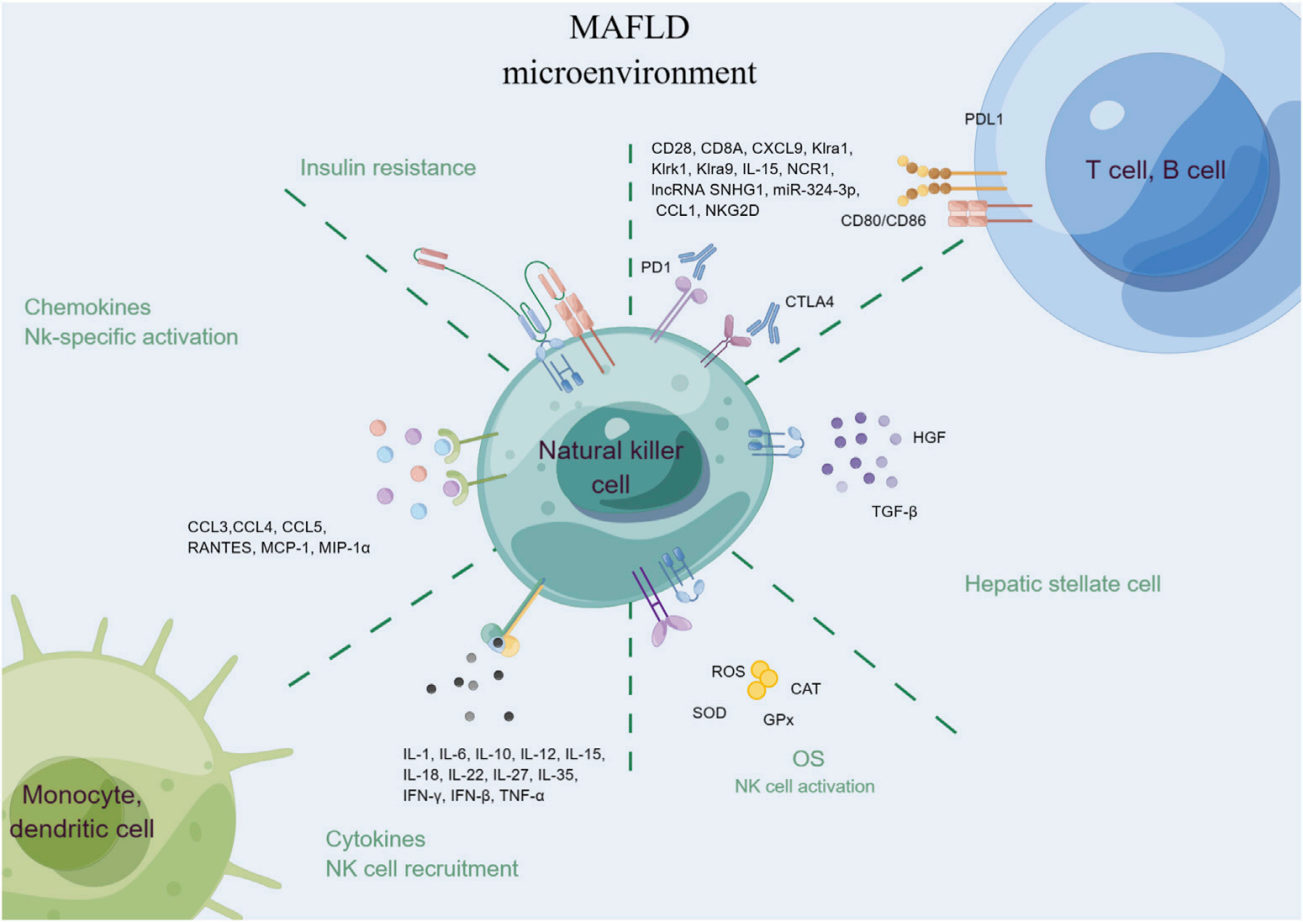
Natural killer cells in MAFLD.

NK cells are innate immune cells that play a role in the early response to viral infections and tumors. They have also been implicated in the development of liver diseases, including MAFLD (Figure 5) (Mendez-Sanchez et al., 2021). NK cells can recognize and kill damaged or infected liver cells, but in MAFLD, their activity may be disrupted, leading to liver injury and inflammation (Abel et al., 2018). For example, NK cells can produce pro-inflammatory cytokines (e.g., IFN-γ, TNF-α, IL-1, IL-6, and IL-12), which can exacerbate hepatic inflammation and promote insulin resistance (Abel et al., 2018). Additionally, NK cells may contribute to the development of liver fibrosis by producing TGF-β and activating HSCs (Fisicaro et al., 2020). Interestingly, recent research has also suggested that NK cells may have a protective role in MAFLD. Studies have shown that increased numbers of NK cells in the liver can lead to improved insulin sensitivity and decreased hepatic steatosis (Wu et al., 2020). Moreover, NK cells may help to clear damaged or infected hepatocytes, and prevent the progression of MAFLD to more severe liver diseases such as cirrhosis and HCC (Sutti and Albano, 2020).

To sum up, while the precise role of NK cells in MAFLD is not yet fully understood, it is clear that they play a crucial role in the development and progression of this disease. Further research is needed to elucidate the complex interplay between NK cells and other immune cells in the liver and to identify potential therapeutic targets for MAFLD.

### 3.6 Dendritic cells

**FIGURE 6:**
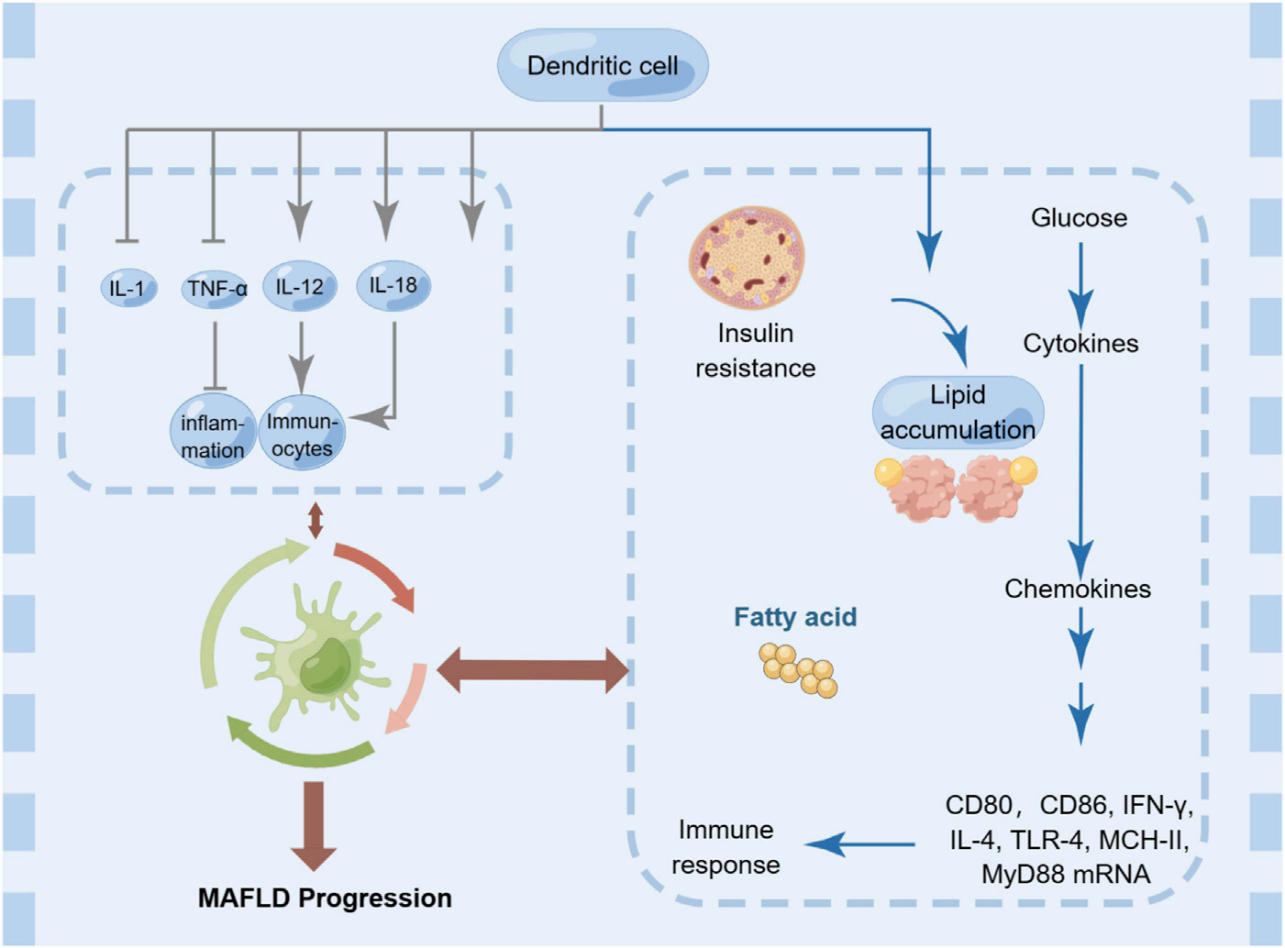
Dendritic cells in MAFLD.

DCs are a type of immune cell that play a crucial role in the regulation of hepatic inflammation and fibrosis, which are key components of the pathogenesis of MAFLD (Figure 6). There is evidence to suggest that DCs can contribute to the progression of MAFLD by promoting inflammation and fibrosis in the liver, as well as by impairing insulin signaling (Ferreyra Solari et al., 2012). In particular, it has been shown that DCs accumulate in the liver of patients with MAFLD and produce pro-inflammatory cytokines (such as IL-1, TNF-α, IL-12, and IL-18) that drive the recruitment and activation of other immune cells, such as macrophages, T cells, and neutrophils (Heier et al., 2017; Yang et al., 2021). Moreover, DCs also contribute to the activation and differentiation of regulatory T (Treg) cells, which play a critical role in suppressing immune responses and maintaining immune tolerance (Wang et al., 2023). Dysregulation of DC function in MAFLD may hinder the induction of Treg cells and promote inflammation and fibrosis. In addition to their antigen-presenting capacity, DCs also produce cytokines and chemokines that influence the recruitment and activation of other immune cells (Chen and Tian, 2020). For example, DC-derived IL-12 and IL-18 promote Th1 and NK cell responses, while DC-derived IL-23 promotes Th17 and γδ T cell responses (Zwirner and Ziblat, 2017). Additionally, DCs can stimulate the differentiation of fibroblasts into myofibroblasts, which are responsible for the excessive deposition of extracellular matrix in the liver, leading to fibrosis (Lakshman et al., 2015). On the other hand, there is also evidence to suggest that DCs can play a protective role in MAFLD by promoting the clearance of lipids from the liver and modulating the immune response to reduce inflammation and fibrosis (Chen and Tian, 2020).

Overall, the relationship between DCs and MAFLD is complex and context-dependent, and DCs represent a promising therapeutic target for the management of this disease. Strategies aimed at modulating DC function, such as using DC-based vaccines or targeting DC-produced cytokines, may help to restore immune homeostasis and alleviate liver inflammation and fibrosis in MAFLD.

### 3.7 Natural killer T (NKT) cells

NKT cells are a subset of T cells that have unique characteristics and play a crucial role in immune surveillance and defense against infections. However, they can also contribute to the development of autoimmune and inflammatory diseases. In MAFLD, NKT cells have been shown to accumulate in the liver and promote inflammation and fibrosis by secreting cytokines and activating other immune cells (Van Herck et al., 2019). Studies have demonstrated that NKT cells are activated in response to lipid accumulation in the liver and contribute to the progression of MAFLD (Gebru et al., 2021). These cells can also recognize and respond to lipid antigens presented by specialized immune cells called antigen-presenting cells. NKT cells can produce pro-inflammatory cytokines like IFN-γ, TNF-α and IL-2, which further activate immune cells and initiate a cascade of events leading to liver damage (Li et al., 2010). Moreover, NKT cells have been implicated in the development of hepatic fibrosis, a significant complication of MAFLD (Chen and Tian, 2020). In animal models of MAFLD, blocking NKT cell activation has been shown to reduce liver inflammation and fibrosis (Chen and Tian, 2020).

Broadly speaking, NKT cells are important players in the pathogenesis of MAFLD, contributing to liver inflammation and fibrosis. Further research is needed to understand the precise mechanisms of NKT cell activation in MAFLD and to identify novel therapeutic targets.

### 3.8 Mast cells

Mast cells and MAFLD have a complex relationship. Mast cells are immune cells that play a significant role in allergic reactions and inflammatory responses, while MAFLD is a metabolic disorder characterized by the accumulation of fat in the liver, which can lead to inflammation, fibrosis, and eventually cirrhosis (Kennedy et al., 2021). Studies have shown that mast cells can contribute to the development and progression of MAFLD. Mast cells release pro-inflammatory cytokines and histamine, which can promote the recruitment of immune cells and cause liver damage (Kennedy et al., 2021). Furthermore, mast cells can stimulate HSCs, which are responsible for producing excess collagen and contributing to liver fibrosis (Kyritsi et al., 2021). On the other hand, some research suggests that mast cells may also play a protective role in MAFLD (Takai and Jin, 2020). Mast cells have been shown to reduce inflammation in certain contexts, and may help clear damaged liver cells (Meadows et al., 2021). Additionally, mast cell activation has been associated with increased adipose tissue browning and improved glucose metabolism in animal models of obesity and diabetes, which are risk factors for MAFLD (Lama et al., 2022).

In general, the relationship between mast cells and MAFLD is complex and may depend on various factors, including the stage and severity of the disease. Further research is needed to fully understand the mechanisms involved and potential therapeutic targets for MAFLD.

## 4 Traditional Chinese medicine in MAFLD

**FIGURE 7:**
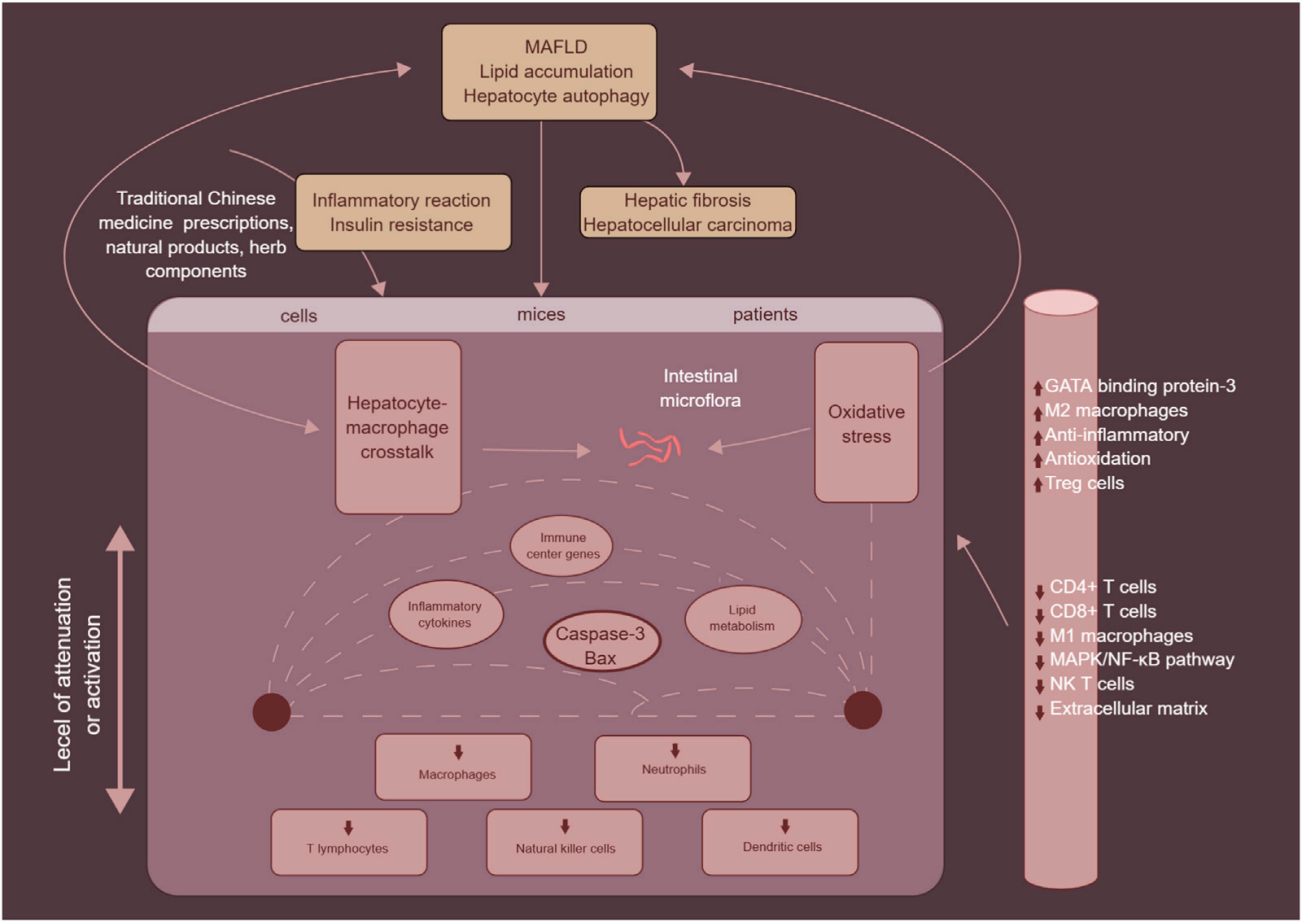
The mechanism of traditional Chinese medicine prescriptions, natural products and herb components in the treatment of MAFLD by targeting immune cells, mainly including macrophages, neutrophils, T cells, NK cells, and DCs.

TCM prescriptions, natural products and herb components, which are primarily sourced from plants, microorganisms, and animals, along with their secondary metabolites, have been found to be valuable in the treatment of various human diseases, owing to their accessibility, applicability, and ability to reduce cytotoxicity. A growing body of evidence supports the use of TCM prescriptions, natural products and herb components as effective approaches for treating MAFLD. They can inhibit the secretion and recruitment of inflammatory factors and cells, regulate liver inflammation and tissue repair, reverse fatty degeneration in MASH, reduce hepatocyte apoptosis and liver fibrosis, and delay the progression of MAFLD (Ma et al., 2021). Recent advances have been made in the development of TCM prescriptions, natural products and herb components for the treatment of MAFLD, with a focus on immune cells such as macrophages, neutrophils, T cells, NK cells, and DCs (as shown in Figure 7). These findings pave the way for the discovery and development of new anti-MAFLD drugs using TCM prescriptions, natural products and herb components.

### 4.1 Target macrophages

TCM prescriptions, natural products and herb components have been shown to impact various aspects of MAFLD pathology, including the secretion and recruitment of macrophages, polarization of M1 to M2 cells, and inhibition of NLRP3 inflammasome activation (Zhang et al., 2020b; Zhou et al., 2021). They can also improve insulin resistance, liver lipid metabolism, and reduce hepatocyte apoptosis, leading to improved liver health and reduced fibrosis (Jeon et al., 2022; Liu et al., 2022). There has been a growing body of research reporting on the effects and mechanisms of TCM prescriptions, natural products and herb components for the treatment of MAFLD, as highlighted in Table 1.

**TABLE 1:** Traditional Chinese medicine prescriptions, natural products and herb components target macrophages to treat MAFLD.

Herb components	Experiment object	Diet	Intervention mode	Mechanism	References
Glycyrrhizin	6-8-week-old male C57BL/6 mice	MCD; MCS	I.p. 2 weeks	Inhibit NLRP3 inflammatory bodies induced by various DAMPs or pathogen-related molecular patterns in macrophages.	Yan et al. (2018)
Hyperoside	C57BL/6 mice	HFD	I.p. 8 weeks	Upregulate NR4A1, promote pro-inflammatory M1 macrophages into anti-inflammatory M2 macrophages, and reduced MAFLD.	Sun et al. (2021)
Cannabinoid Abn-CBD	10-week-old C57Bl/6J mice	HFD	I.p. 2 weeks	Impair macrophage infiltration, reduce apoptosis, and eliminate liver inflammation and fibrosis.	Romero-Zerbo et al. (2020)
Curcumin	Male C57BL/6 mice, RAW264.7 cell	MCD	Gavage 8 weeks	Inhibit M1 macrophages. and secretion of inflammatory.	Tong et al. (2021)
Gallic acid	HepG2, murine hepatoma cell line Hepa, and murine macrophage cell line RAW 264	PA	Gavage 24 h	Downregulate MAPK/NF-κB.	Tanaka et al. (2020)
Epigallocatechin-3-gallate (EGCG)	8-week-old male C57BL/6J mice	HFD plus fructose	Oral gavage 8 weeks	Promote M1 to M2, reduce the secretion of inflammatory mediators, reduce liver.	Du et al. (2021)
Cordycepin (CRD)	8–10-week-old male C57BL/6J mice	HFD	Gavage 8 weeks	Reduce inflammatory factors.	Gong et al. (2021)
Rhodiola	8-week-old male C57BL/6J mice, HepG2 cell	HFD	Gavage 16 weeks	Activate macrophage migration inhibitor and relieve MAFLD.	Liu et al. (2022)
Glucoraphanin	Male C57BL/6JSlc mice	HFD	Feed 14 weeks	Macrophage accumulation and M2 polarity of liver and fat macrophages, and improve liver steatosis and insulin resistance.	Nagata et al. (2017)
Honokiol	7-8-week-old male C57BL/6J mice	CL; HFD	Feed 12 weeks	Polarize macrophages into M2 phenotype and improve MASH.	Zhong and Liu (2018)
Glycyrrhetinic acid	8-week-old male C57BL/6 mice	HFD plus fructose	Intragastric administration 2 weeks	Regulate the activation of macrophages, improve the damaged autophagy flux, reduce the excessive production of inflammatory cytokines, and improve the excessive apoptosis of hepatocytes, thus playing a therapeutic role in MAFLD.	Fan et al. (2022)
Vitexin	Male C57BL/6 mice	HFD	Feed 5 weeks	Significantly reduce the infiltration of hepatic macrophages, significantly downregulated the mRNA and protein expression of hepatic SREBP-1c, FAS and ACC, inhibit the signal transduction of TLR4/NF-κB, reduce fatty acid synthesis protein, and improved MAFLD.	Li et al. (2020)
Resveratrol	Male C57BL/6 mice, 293T cell	MCD	Intragastric administration 4 weeks	Prevent liver cell damage induced by inflammatory cytokines released by foam macrophages, and inhibit the development of MASH.	Che et al. (2020)
Andrographolide (ANDRO)	10-week-old male C57 BL/6 mice	CDAA	I.p. 22 weeks	Significantly reduce the infiltration of liver macrophages and promote the mRNA level of liver pro-inflammatory and pro-fibrosis genes.	Cabrera et al. (2017)
Rapeseed protein hydrolysates	Male C57BL/6J mice	HFD	I.p. 6 weeks	Inhibit macrophages infiltration induce liver pro-inflammatory and fibrosis genes expression.	Zhao et al. (2019)
Sugar kelp	7-week-old male C57BL/6J mice	HFD; high-sucrose; high-cholesterol diet	Feed 14 weeks	Reduce the expression of macrophage marker adhesion G protein-coupled receptor E1 (ADGRE1) and M1 macrophage marker integrin α x (ITGAX), alleviate the inflammatory reaction and improve the liver steatosis.	Kim et al. (2021)
Tetrahydrocurcumin (THC)	4-week-old male C57BL/6J mice	HFD	Oral gavage 10 weeks	Reduce macrophage recruitment, liver inflammation and cytokines in adipose tissue, and improve insulin resistance and liver steatosis.	Pan et al. (2018)
Geniposide and chlorogenic acid (GC)	6-week-old male C57BL/6 mice	HFD	Feed 5 weeks	Inhibit macrophage activation to improve inflammation and achieve the purpose of treating MASH’s steatosis.	Xin et al. (2021)
Lycopene	7-week-old male C57BL/6J mice	CL plus HFD	Feed 12 weeks	Regulate LPS-/IFN-γ/TNFα in peritoneal macrophages also the expression of fiber gene, and reverse inflammation and fibrosis induced by lipid toxicity in MASH mice.	Ni et al. (2020)
β-cryptoxanthin	8-week-old C57BL/6J mice	CL plus HFD	Feed 2 weeks	Downregulate M1-labeled mRNA induced by lipopolysaccharide in peritoneal macrophages, promote the expression of M2-labeled mRNA induced by IL-4, and alleviate insulin resistance and steatohepatitis.	Ni et al. (2015a)
An organic extraction from lemon balm	HUVECs、10-week-old C57BL/6J mice	HFD	Feed 15 weeks	Inhibit the expression of inflammatory marker genes such as TNF-α, CD68 and MCP-1, reduce macrophages and inhibit the secretion of inflammatory cytokines.	(Kim et al., 2017a)
Astaxanthin (ASTX)	8-week-old male C57BL/6J mice	HFD	Feed 18 weeks	Reduce the infiltration of macrophages and the expression of M1 macrophage markers, and inhibit the inflammation and fibrosis in the liver and adipose tissue of obese mice.	Kim et al. (2017b)
Naringenin	2-month-old male wistar rat	HCD	Oral gavage 3 months	Decrease the expression of mucin-like hormone receptor-like 1 (specific gene of macrophage F4/80), regulate the level of necrotizing inflammation, promote the degradation of extracellular matrix, and prevent MASH and fatty fibrosis induced by cholesterol in rats.	Chtourou et al. (2015)
An extract of O ficus-indica seed	Male C57BL/6 mice	HFD	Oral gavage 4 weeks	Regulate liver macrophage polarization and adipogenesis to improve liver steatosis and inflammation, so as to combat experimental MAFLD.	Kang et al. (2016)
Inulin (INU)	4-week-old male C57BL/6 mice	HFD	Oral gavage 14 weeks	Suppress liver macrophages to alleviate the inflammatory reaction of the liver and prevent MAFLD.	Bao et al. (2020)
Baicalin (BA)	6-week-old male C57BL/6J mice	MCD	Oral gavage 4 weeks	Alleviate liver inflammation, which is related to the inhibition of macrophage influx and NF-κB activation.	Zhang et al. (2018)
Yinzhihuang (YZH)	8-week-old male C57BL/6J mice	HFD	Oral gavage 16 weeks	Reduce the infiltration of macrophages, especially the infiltration of pro-inflammatory M1, and inhibite the pathways of TLR4, Myeloid differentiation primary response gene 88 (MyD88) to prevent MASH.	Li et al. (2022)
Emodin	6-week-old female LDLR/mice	HFD	I.p. 4 weeks	Reduce the infiltration of macrophages and granulocytes in the liver, inhibit systemic and local inflammatory reactions in the liver, and inhibit the transition from simple steatosis to MASH.	Jia et al. (2014)
Myricetin	6-week-old male C57BL/6J mice, RAW264.7 cell	CDAHFD	Oral gavage 8 weeks	Regulate the polarization of macrophages, thus alleviating MASH and liver fibrosis.	Yao et al. (2020)
Boccoliwater	6-week-old male C57BL/6J mice	MCD; MCS	Gavage 8 weeks	Regulate the M1/M2 polarization of macrophages, inhibit the inflammation of MAFLD, and delay the occurrence and development of MAFLD.	Huang et al. (2022)
Curcumin	4-week-old male C57BL/6J mice	HFD	Feed 24 weeks	Inhibit the accumulation of macrophages in liver and improve MAFLD.	Inzaugarat et al. (2017)
Limonin	Zebrafish	Fertilized embryos	Exposure 72 h	Inhibit the infiltration of macrophages, downregulate the relative expression levels of pro-inflammatory factors IL-6, IL-1β and TNF-α secreted by macrophages, upregulate the NRF2/HO-1 signal pathway in the liver to reverse the reduction of glutathione and the accumulation of ROS, and exert its resistance to lipid deposition, antioxidant and anti-inflammatory effects to protect MAFLD.	Li et al. (2021b)
RFAs	C57BL/6 mice, mouse primary hepatocyte	MCD	Gavage 3 weeks	Inhibit NLRP3 inflammatory bodies and liver Kuffer cells to improve MAFLD.	Wu et al. (2022b)
YCHD	C57BL/6 mice, mouse primary hepatocytes, Kuffer cell	MCD	Gavage 9 weeks	Inhibit NLRP3 inflammatory bodies and liver Kuffer cells to improve MAFLD.	Wu et al. (2022b)
Apigenin (API)	4–6 week-old male C57BL/6 J	HFD	Gavage 4 weeks	Regulate the recruitment of macrophages, inhibit inflammation, regulate liver lipid metabolism, and inhibit liver steatosis.	Lv et al. (2019)

Abbreviations: i. p, intraperitoneal injection; MCD, Methionine- and choline-deficient diet; MCS, the methionine- and choline-sufficient diet; CDAA, Choline-deficient amino acid-defined diet; PA, palmitic acid; CDAHFD, Choline-deficient, L-amino acid-defined, high-fat diet.

For example, a triterpene glycoside called glycyrrhizin (GL), which is commonly used as a food sweetener or active pharmaceutical ingredient, possesses a range of medicinal properties such as anti-ulcer, anti-spasm, anti-inflammatory, anti-oxidative, anti-viral, anti-microbial, anti-cancer, and anti-androgen properties (Eisenbrand, 2006). Research suggests that GL has potential as a therapeutic agent for MAFLD due to its ability to inhibit NLRP3 inflammasome activation and adipose tissue inflammation, as well as improve insulin sensitivity and reduce liver inflammation and fibrosis in animal models (Chen et al., 2018; Yan et al., 2018; Li et al., 2021a). Therefore, GL could be a promising natural treatment option for MAFLD. Similarly, hypericin, also known as quercetin 3-o-β-d-galactoside, is a bioactive flavonoid glycoside that can be found in Epilobium, Hypericum, and Hypericum (Wu et al., 2023). Numerous studies have demonstrated the wide range of pharmacological activities associated with hypericin, indicating its potential use as a pharmaceutical ingredient (Jiang et al., 2013). Such activities include its antioxidative, hypoglycemic, anti-inflammatory, and anticancer effects. Recent research has shown that hyprepin, which contains hypericin, has the ability to significantly improve hepatic steatosis, insulin resistance, and inflammatory response in liver tissue from C57BL/6 mice treated with hyprepin (Sun et al., 2021). It also modulated macrophage polarization, which was found to be dependent on the nuclear receptor subfamily 4 group a member 1 (NR4A1) (Sun et al., 2021). This highlights the therapeutic potential of hypericin in regulating macrophages and preventing the pathological progression of MAFLD (Sun et al., 2021). There is evidence to suggest that the cannabinoid Abn-CBD can have beneficial effects on the liver by impairing macrophage infiltration, reducing apoptosis, and eliminating liver inflammation and fibrosis (Romero-Zerbo et al., 2020). Some studies have shown that Abn-CBD can reduce the levels of pro-inflammatory cytokines and chemokines, which are responsible for attracting immune cells to the liver and promoting inflammation (Romero-Zerbo et al., 2020). Additionally, Abn-CBD has been shown to activate the CB2 receptor, which is expressed on immune cells such as macrophages, leading to a suppression of their activity and infiltration into the liver (Romero-Zerbo et al., 2020). This can result in a reduction in liver inflammation and fibrosis (Romero-Zerbo et al., 2020). The ability of Abn-CBD to reduce apoptosis, or programmed cell death, in liver cells may also contribute to its therapeutic potential for MAFLD (Romero-Zerbo et al., 2020). However, further research is needed to fully understand the mechanisms underlying the effects of Abn-CBD on MAFLD and to determine its potential as a therapeutic agent.

By and large, the research suggests that TCM prescriptions, natural products, and herb components have the potential to be effective treatments for MAFLD through their modulation of macrophage activity and inflammation. However, more studies are needed to identify the optimal composition, concentration, and dosage of these treatments, as well as to better understand their mechanisms of action.

### 4.2 Target neutrophils

TCM prescriptions, natural products, and herb components have been found to play a significant role in the anti-MAFLD process by regulating the activation, infiltration, and metabolism of neutrophils. This helps in controlling liver inflammation, hepatocyte apoptosis, liver injury, and the degree of liver lipid accumulation, as highlighted in Table 2.

**TABLE 2:** Herb components and natural products target neutrophils to treat MAFLD.

Herb components	Experiment object	Diet	Intervention mode	Mechanism	References
Berberine (BBR)	8–12-weeks-old male and female mice	HFD plus high-fructose diet	Oral gavage 9 weeks	Reduce immune cells infiltration, inhibit neutrophils activation, as well as decreasing inflammatory genes expression to significantly inhibit inflammation and improve MAFLD.	Wang et al. (2021b)
Resveratrol; quercetin	25-days-old male Wistar rat	30% sugar	Feed 4 weeks	Downregulate neutrophils elastase; reduce hepatocyte apoptosis and hepatic fibrosis.	Cano-Martinez et al. (2021)
Tanshinone IIA (TIIA)	7-week-old female C57BL/6 mice	MCD	I.p. 6 weeks	Inhibit the formation of myeloperoxidase (MPO) and citrullinated histone H3 (CITH3) in NETs, and inhibit the apoptosis of hepatocytes mediated by caspase-3 and bax, thus alleviating the liver inflammatory response.	Xu et al. (2022)
Baicalin	6–8-week-old male C57BL/6J mice	MCD	I.p. 4 weeks	Reduce the infiltration of neutrophils and macrophages, reduce liver inflammation, reduce hepatocyte apoptosis and liver injury, reduce liver lipid accumulation, reduce liver fibrosis and improve MAFLD.	Liu et al. (2020)
Hazelnut oil	5-week-old male hamster	HC	Feed	Reduce neutrophils infiltration in MASH, reduce glycogen accumulation in liver, and inhibit liver inflammation and fibrosis.	Lu et al. (2019)
Quercetin	Sprague-Dawley rat	HFD	Feed 8 weeks	Reduce neutrophils infiltration and lymphocytes infiltration, inhibit liver inflammation and steatosis, and delay the progress of MAFLD.	Zhou et al. (2013)

Berberine (BBR), an isoquinoline-like quaternary alkaloid extracted mainly from Coptis chinensis Franch, has been found to have beneficial effects on various metabolic diseases, including T2DM, obesity, MAFLD, hyperlipidemia, and gout, based on animal studies (Imenshahidi and Hosseinzadeh, 2016; Xu et al., 2021). BBR has shown promising results in improving hepatic steatosis and reducing serum LDL cholesterol levels (Hu et al., 2022). Additionally, it has been observed that BBR can alleviate liver fibrosis by reducing the infiltration of immune cells, inhibiting neutrophil activation and the expression of inflammatory genes, and regulating the expression of multiple genes that are involved in HSCs activation and bile duct cell proliferation (Wang et al., 2021b). As such, BBR holds great potential as a therapeutic agent for MAFLD (Wang et al., 2021b). Resveratrol and quercetin are two natural compounds that have been shown to exert beneficial effects on various aspects of health (Tian and Liu, 2020). One of their potential mechanisms of action is down-regulating neutrophil elastase, an enzyme involved in the breakdown of the extracellular matrix in tissues (Cano-Martinez et al., 2021). Neutrophil elastase is released by neutrophils, a type of white blood cell, in response to inflammation and tissue damage (Cano-Martinez et al., 2021). While it plays an important role in fighting infections, excessive production of neutrophil elastase can lead to tissue destruction, especially in chronic inflammatory conditions (Cano-Martinez et al., 2021). Resveratrol and quercetin have been found to inhibit neutrophil elastase activity, which may help prevent tissue damage and reduce inflammation in various organs, including the liver (Cano-Martinez et al., 2021). In fact, studies have shown that these compounds can reduce hepatocyte apoptosis (cell death) and hepatic fibrosis (excessive scar tissue formation) in animal models of MAFLD (Cano-Martinez et al., 2021). In addition, tanshinone IIA (TIIA) is a natural compound found in the roots of Salvia miltiorrhiza, a traditional Chinese herb (Subedi and Gaire, 2021). Research has shown that TIIA has anti-inflammatory and anti-oxidative effects, making it a potential therapeutic agent for inflammatory liver diseases (Xuan et al., 2017). Neutrophil extracellular traps (NETs) are web-like structures composed of DNA, histones, and granule proteins that are released by neutrophils during inflammation (Yang et al., 2020). MPO and CITH3 are two components of NETs that contribute to the pro-inflammatory response (Saisorn et al., 2021). TIIA has been shown to inhibit the formation of MPO and CITH3 in NETs, thereby reducing the inflammatory response in the liver (Xu et al., 2022). Caspase-3 and bax are two proteins involved in the apoptotic pathway in cells (Xu et al., 2022). Inflammatory liver diseases often lead to hepatocyte apoptosis (Xu et al., 2022). TIIA has been shown to inhibit caspase-3 and bax-mediated apoptosis of hepatocytes, thereby preventing liver damage and inflammation (Xu et al., 2022). Overall, TIIA’s ability to inhibit NET formation and hepatocyte apoptosis make it a promising therapeutic agent for MAFLD (Xu et al., 2022).

Generally, these herb components and natural products have shown promising effects in the treatment of MAFLD by regulating neutrophils involved in its pathogenesis. However, further studies are needed to confirm their efficacy and safety in humans before they can be recommended for clinical use.

### 4.3 Target T lymphocytes

Popular herb components and TCM prescriptions, including astaxanthin, β-cryptoxanthin, theaphenon E (TE), curcumin, rhein, and qushi huayu decoction, have shown promise for the prevention and treatment of MAFLD/MASH. Through cellular or animal experiments, these natural substances have been observed to target T cells and effectively treat MAFLD (Table 3).

**TABLE 3:** Traditional Chinese medicine prescriptions, natural products and herb components target T cells to treat MAFLD.

Herb components	Experiment object	Diet	Intervention mode	Mechanism	References
Astaxanthin	7-week-oldmale C57BL/6J mice, 5-week-old male ob/ob mice	HFD	Feed 10 weeks	Inhibit the CD4^+^ and CD8^+^ T cells recruitment in liver, promote M2 macrophages, reduce liver inflammation, fibrosis, and prevent MASH.	Ni et al. (2015b)
β-cryptoxanthin	8-week-old C57BL/6J mice	CL	Feed 2 weeks	Inhibit the CD4^+^ and CD8^+^ T cells recruitment in liver, improve steatosis, inflammation and fibrosis in MASH progression.	Ni et al. (2015a)
Theaphenon E (TE)	Male C57BL/6J mice, primary human liver cells	HFD	Feed 35 weeks	Regulate CD4^+^ T cells viability; induce apoptosis, inhibit the lipid accumulation of MAFLD.	Coia et al. (2021)
Curcumin	4-week-old male C57BL/6J mice	HFD	Feed 24 weeks	Monocytes accumulation, improve liver histological function in MAFLD.	Inzaugarat et al. (2017)
Rhein	10 to 12-week-old female C57BL/6J mice, GW-3965	HFD	Gavage 40 days	Inhibit T-box (T-bet) in T cells and increase the activation of transcription 6 (STAT6) phosphorylation, thus regulating the Th1/Th2 response, inhibiting pro-inflammatory cytokines expression and reversing hepatic steatosis.	Sheng et al. (2011)
Qu shi hua yu decoction	4-week-old male Sprague-Dawley rats or C57BL/6J mice	CL	Gavage 4 weeks	Promote the function of Tregs induced intestinal microflora, and decreased the synthesis of MASH.	Feng et al. (2017)

Abbreviations: HFD, high-fat diet; CL, High-cholesterol diet.

For example, astaxanthin, a ketocarotenoid with the chemical name 3, 3′-dihydroxy-4, 4′-diketonyl-β, β′-carotene, can directly enter cells and quench ROS and free radicals, allowing it to function as a natural antioxidant, which is 500 times more active than vitamin E (Ambati et al., 2014). Astaxanthin is known to show a wide range of beneficial effects, involving anti-inflammatory and antitumor activities (Jia et al., 2016; Kim et al., 2017b). Astaxanthin reduces the recruitment of CD4^+^ and CD8^+^ T cells in the liver, reverses insulin resistance, and liver inflammation and fibrosis, and has been shown more effective than vitamin E in the prevention and treatment of MASH (Ni et al., 2015b). Therefore, astaxanthin is particularly promising as a drug for MASH in resource-limited settings, such as underdeveloped countries. In addition, β-cryptoxanthin (other names: β, β-carotene-3-ol), a precursor carotenoid, is widely found in paprika, pumpkin, persimmon, orange, papaya, and peach (Ni et al., 2016). Several potential medicinal values of β-cryptoxanthin have been revealed, such as anti-MAFLD, antioxidant, cancer prevention, and anti-metabolic syndrome (Burri, 2015; Hirata et al., 2019). Application of β-cryptoxanthin helps to inhibit the recruitment of CD4^+^ and CD8^+^ T cells in the liver, attenuate insulin resistance and excessive lipid accumulation and peroxidation in the liver, and ultimately prevent or reverse inflammation and fibrosis in MASH (Ni et al., 2015a). Several other natural compounds with regulating T cells and anti-inflammatory properties have been studied for their potential use in MAFLD, including theaphenon E (TE) (Coia et al., 2021), curcumin (Inzaugarat et al., 2017), rhein (Sheng et al., 2011), and qu shi hua yu decoction (Feng et al., 2017).

However, the bioavailability and stability of TCM prescriptions and herb components *in vivo* are limited, due to its poor solubility and rapid metabolism and elimination. Therefore, various strategies have been developed to enhance the delivery and efficacy of TCM prescriptions and herb components, such as using nanoparticles, liposomes, phospholipid complexes, or analogs of TCM prescriptions and herb components with improved pharmacokinetic properties.

### 4.4 Target natural killer cells

The treatment of MAFLD has long been a focus of TCM prescriptions, natural products, and herb components research and has captured the attention of researchers worldwide. Korean red ginseng (KRG), urushiol, and qiang gan formula have been extensively studied for their potential therapeutic benefits in addressing MAFLD. In fact, studies have shown that KRG, urushiol and qiang gan formula may have a positive impact on NK cell activity in a mouse model of MAFLD.

The KRG herb has been found to have multiple pharmacological effects on immune deficiency, metabolic syndrome, and cancer (Kim et al., 2013). Urushiol, derived from Rhus vernicifera plants, has been shown to inhibit the growth of ovarian cancer, murine leukemia, and human adenocarcinoma (Suk et al., 2010). Additionally, the mechanism of inhibition of MAFLD by KRG and urea is related to its anti-fibrotic and antioxidant mechanisms by inhibiting NK cell expression in rats (Hong et al., 2013). Qiang gan, a traditional Chinese medicine formula consisting of 16 herbs, has also been reported to be effective in treating MAFLD/MASH, with enhanced NK cell activity and improved hepatic steatosis and inflammation in mice. This formula works by inhibiting NK cell-mediated cytotoxicity, eliminating the pro-inflammatory state of fatty liver, reducing hepatocyte inflammation, and improving lipid metabolism (Zhu et al., 2019).

Despite promising results from preclinical studies, further research is needed to determine the safety and efficacy of these TCM prescriptions, natural products, and herb components in treating MAFLD in humans. Additionally, the use of these herbs should be supervised by a qualified healthcare provider, as some may interact with medications or have potential side effects.

### 4.5 Target dendritic cells

In addition to the previously mentioned TCM prescriptions, natural products, and herb components, several other drugs have demonstrated potential therapeutic effects on MAFLD by mediating DCs. The Jiang Zhi Granule (JZG), composed of main compounds from Salvia miltiorrhiza Bunge (Lamiaceae), Folium nelumbinis, Polygala tenuifolia Willd. (Polygalaceae), Artemisia capillaris Thunb. (Asteraceae), and Gynostemma pentaphyllum (Thunb.) Makino (Cucurbitaceae), has been reported to be effective against MAFLD (Yu et al., 2021). Additionally, studies have shown that JZG can promote the maturation of intestinal mucosal DCs and induce the differentiation of immature CD4^+^ T cells into Th1 cells (Yu et al., 2021). It can also reduce damage to the intestinal mucosal immune barrier and decrease liver fat in MASH rats, resulting in significant improvements in liver function and liver tissue pathology (Yu et al., 2021).

On the whole, the TCM prescription have been reported to have potential therapeutic effects on MAFLD by mediating DCs. The treatment can improve liver function and decrease liver fat accumulation by reducing inflammation and inhibiting lipid accumulation in hepatocytes. Further studies are needed to investigate the underlying mechanisms of these treatments and to develop effective therapies for MAFLD.

## 5 Discussion

MAFLD encompasses a range of liver diseases, such as MASH which is characterized by the accumulation of fat in the liver. Its core histopathological features include inflammation, hepatocyte damage, and varying levels of fibrosis. The global prevalence of MAFLD is on the rise, but unfortunately, there are currently no FDA-approved medications to treat this condition. The primary treatment for MAFLD is weight loss through dietary and lifestyle modifications, managing metabolic risk factors, and drug therapy. However, these treatments have not produced satisfactory results. In the liver, immune cells, both innate and adaptive, tend to exhibit a pro-inflammatory phenotype in MAFLD and MASH and play a significant role in driving the progression of these diseases. Targeting liver immune cells presents a promising strategy for effective treatment of MAFLD/MASH.

According to statistics from the World Health Organization (WHO), a significant portion of the global population in developing countries relies on TCM prescriptions, natural products, and herb components for primary healthcare. Furthermore, combining TCM with clinical medicines can enhance the curative effect of conventional medicine, mitigate its side effects, and improve the patient’s survival rates and quality of life. Recently, there has been growing interest in exploring the therapeutic potential of TCM prescriptions, natural products, and herb components in treating MAFLD. Therefore, this review aims to investigate the effectiveness of TCM prescriptions, natural products, and herb components as potential treatments for MAFLD.

The therapeutic efficacy of TCM prescriptions, natural products, and herb components in the treatment of MAFLD lies primarily in their ability to regulate immune cell infiltration (including macrophages, neutrophils, DCs, T cells and NK cells), which in turn inhibits the secretion and recruitment of inflammatory factors and cells, as well as regulates hepatic inflammatory responses and tissue repair. These interventions can also reverse fatty degeneration in MASH, reduce hepatocyte apoptosis, and alleviate liver fibrosis, ultimately slowing down the progression of MAFLD. Nevertheless, few clinical reports are available on the use of TCM prescriptions, natural products, and herb components in immune cell-targeted treatment of MAFLD. In this review, we summarize and analyze existing evidence of TCM prescriptions, natural products, and herb components that mediate immune cells to improve MAFLD, providing new guidance for the clinical search for new anti-MAFLD targets and the development of new anti-MAFLD drugs.

However, despite the potential benefits of TCM prescriptions, natural products, and herb components, the application of these treatments in clinical practice is still fraught with difficulties. For instance, it remains unclear whether some natural products can be safely used as clinical drugs or dietary supplements. To better understand the therapeutic effects of TCM prescriptions, natural products, and herb components on MAFLD, further in-depth studies are required. Additionally, while certain active ingredients may have greater efficacy when used in combination with other drugs, their use is often limited due to poor water solubility and low bioavailability, making it challenging to modulate immune cells against MAFLD. Lastly, most studies conducted thus far have been limited to cellular and animal models, underscoring the need for more extensive clinical trials to establish their reliability.

Another challenge in using TCM prescriptions, natural products, and herb components is the lack of standardization and quality control. The composition and concentration of active ingredients can vary widely depending on factors such as the source, processing, and storage of the raw materials. This inconsistency may lead to variable therapeutic effects and unpredictable adverse reactions. Moreover, there is also a need for proper regulation and oversight of the production and marketing of TCM prescriptions, natural products, and herb components. The absence of appropriate regulations may lead to substandard products and misleading claims about their efficacy and safety. To overcome these challenges, more rigorous research is needed to develop evidence-based guidelines for the clinical use of TCM prescriptions, natural products, and herb components. This includes better characterization and standardization of active ingredients, as well as clinical trials to evaluate their safety and efficacy. In addition, there should be closer collaboration between traditional medicine practitioners and mainstream healthcare professionals to ensure coordinated and integrated care for patients with MAFLD.
